# Local Antibiotic Delivery Ceramic Bone Substitutes for the Treatment of Infected Bone Cavities and Bone Regeneration: A Systematic Review on What We Have Learned from Animal Models

**DOI:** 10.3390/ma16062387

**Published:** 2023-03-16

**Authors:** Nuno Alegrete, Susana R. Sousa, Bárbara Peleteiro, Fernando J. Monteiro, Manuel Gutierres

**Affiliations:** 1 i3S-Instituto de Investigação e Inovação em Saúde, Universidade do Porto, R. Alfredo Allen 208, 4200-135 Porto, Portugal; 2 INEB-Instituto de Engenharia Biomédica, R. Alfredo Allen 208, 4200-135 Porto, Portugal; 3 FMUP-Faculdade de Medicina, Universidade do Porto, Alameda Prof. Hernâni Monteiro, 4200-319 Porto, Portugal; 4 ISEP-Instituto Superior de Engenharia do Porto, IPP - Instituto Politécnico do Porto, R. Dr. António Bernardino de Almeida 431, 4200-072 Porto, Portugal; 5 EPIUnit-Instituto de Saúde Pública, Universidade do Porto, Rua das Taipas 135, 4050-600 Porto, Portugal; 6 Departamento de Ciências da Saúde Pública e Forenses e Educação Médica, Faculdade de Medicina, Universidade do Porto, Alameda Prof. Hernâni Monteiro, 4200-319 Porto, Portugal; 7 ITR-Laboratório para a Investigação Integrativa e Translacional em Saúde Populacional, Rua das Taipas 135, 4050-600 Porto, Portugal; 8 FEUP-Faculdade de Engenharia, Universidade do Porto, R. Dr. Roberto Frias, 4200-465 Porto, Portugal; 9 CHUSJ-Centro Hospitalar Universitário S. João, Alameda Prof. Hernâni Monteiro, 4200-319 Porto, Portugal

**Keywords:** ceramic-based biomaterials, scaffolds, bone regeneration, bone-graft substitutes, bone infection, antibacterial materials

## Abstract

Aims: the focus of this study is to evaluate if the combination of an antibiotic with a ceramic biomaterial is effective in treating osteomyelitis in an infected animal model and to define which model and protocol are best suited for in vivo experiments of local bone infection treatment. Methods: a systematic review was carried out based on PRISMA statement guidelines. A PubMed search was conducted to find original papers on animal models of bone infections using local antibiotic delivery systems with the characteristics of bone substitutes. Articles without a control group, differing from the experimental group only by the addition of antibiotics to the bone substitute, were excluded. Results: a total of 1185 records were retrieved, and after a three-step selection, 34 papers were included. Six manuscripts studied the effect of antibiotic-loaded biomaterials on bone infection prevention. Five articles studied infection in the presence of foreign bodies. In all but one, the combination of an antibiotic with bioceramic bone substitutes tended to prevent or cure bone infection while promoting biomaterial osteointegration. Conclusions: this systematic review shows that the combination of antibiotics with bioceramic bone substitutes may be appropriate to treat bone infection when applied locally. The variability of the animal models, time to develop an infection, antibiotic used, way of carrying and releasing antibiotics, type of ceramic material, and endpoints limits the conclusions on the ideal therapy, enhancing the need for consistent models and guidelines to develop an adequate combination of material and antimicrobial agent leading to an effective human application.

## 1. Introduction

The current protocols to treat chronic osteomyelitis consist of the intravenous and oral administration of drugs for long periods and surgical debridement of all devitalized bone fragments [1]. Adequate debridement may leave a bone defect (“dead space”) where achieved antibiotic concentrations are low and require being filled during a second surgery [2,3]. Several strategies to achieve adequate local antibiotic concentrations and fill dead space have been developed. Local antibiotic therapy should provide considerable advantages over the systemic use of antibiotics [4].

The local delivery of antibiotics was introduced into orthopedic surgery by antibiotic-loaded polymetilmetacrylate (PMMA) beads, where the major drawbacks are the required removal and the prolonged delivery of sub-therapeutic antibiotic concentrations [5]. Biodegradable antibiotic-loaded implants may be able to provide adequate local bactericidal tissue concentrations. Different drug-delivery systems (lactic acid and polyglycolic acid) have been investigated [6,7,8,9,10,11], but most have failed to provide the sustained release of the antibiotic at a uniform rate for the required time length [12]; even after the eradication of infection, the problem of dead space is still unsolved.

Bioactive ceramics are suitable bone substitutes due to their biocompatibility, bioactivity, biodegradability, and osteoconductivity [13,14,15,16,17]; when implanted in vivo, they do not induce toxicity or antigenic response [18]. The impregnation of osteoconductive materials (calcium sulfate, hydroxyapatite, and tricalcium phosphate) with antibiotics for the local treatment of osteomyelitis has been considered promising, solving the problem of dead space [19,20,21,22,23] while eradicating infection [24]. Research is ongoing to develop bioceramics that can release antibiotics for a period long enough to treat the infection but that absolutely stop the drug delivery at a certain time point to avoid a low antibiotic concentration and the emergence of bacterial resistance.

The main aim of this review is to evaluate if the combination of an antibiotic with a ceramic biomaterial in an infected animal model is an effective treatment for osteomyelitis. The secondary aims are: first, to define the ideal animal model for the study of antibiotic-releasing ceramic biomaterials; and second, to understand which are the ideal endpoints and methods to evaluate the cure of infection and osseointegration.

## 2. Materials and Methods

**Figure 1:**
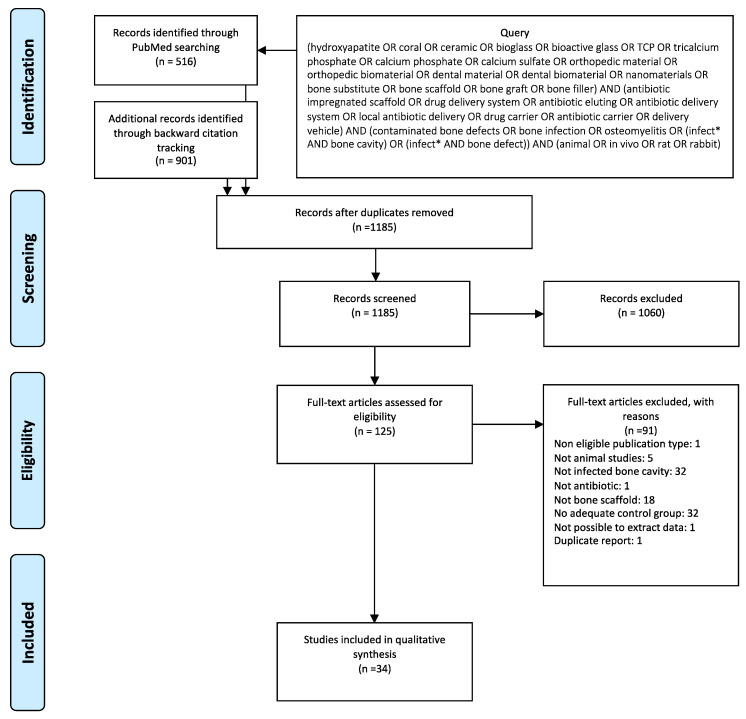
Article selection process. * The truncation symbol (asterisk) was used to search for multiple variants of the word (singular/plural/conjugations, etc.) all at once, e.g., infected, infection, infectious, according to the PubMed search engine guide.

This systematic review was based on PRISMA (Preferred Reporting Items for Systematic Reviews and Meta-Analysis) statement guidelines [25]. Literature search: to identify all in vivo studies with original data on local antibiotic delivery ceramic bone substitutes to treat infected bone cavities, a PubMed search was performed using a combination of the following terms or equivalents: “ceramic bone substitute”, “antibiotic”, “osteomyelitis”, and “animal” (Figure 1). Article selection: study selection was conducted in three steps. In step 1, two researchers screened titles and abstracts independently (N.A. and S.R.S.). In step 2, full-text articles were analyzed independently, and disagreements were discussed between reviewers. When a consensus was not reached, a third researcher was involved in the discussion (F.J.M.). In step 3, data were extracted and analyzed. The exclusion criteria were (1) articles not written in English, Portuguese, or Spanish; (2) article type: editorial, comment, guidelines, case report, abstract, review, or letter; (3) studies not involving animals; (4) studies not dealing with infected bone cavities; (5) studies not using antibiotics; (6) studies where the biomaterial was not ceramic; (7) absence of a control group that received the same biomaterial without antibiotic; (8) non-extractable data; and (9) data duplicated from another article. After the first selection, all cited references of the selected papers were cross-checked, and the screening procedure was repeated. The general results on implant effect were retrieved from individual papers, with data presented according to the causative agent of bone infection, type and composition of each biomaterial, type and amount of antibiotic, time between material implantation and analysis, and presence of concomitant conditions. The reported biomaterial effects on infection and bone remodeling were converted into graphic summary tables. The selected papers’ quality was evaluated by the application of the ARRIVE (Animal Research: Reporting of In Vivo Experiments) guidelines for reporting animal research [26].

## 3. Results

### 3.1. Systematic Review

Five hundred sixteen references were retrieved after a PubMed literature search. After applying the exclusion criteria, a total of 32 articles were included in this review [19,27,28,29,30,31,32,33,34,35,36,37,38,39,40,41,42,43,44,45,46,47,48,49,50,51,52,53,54,55,56,57]. Two additional papers [58,59] were added to the final literature review after backward citation tracking, making a total of 34 papers.

Figure 1 summarizes the article selection process [25].

### 3.2. Sample and Methods of the Selected Studies

#### 3.2.1. Experimental Groups

In 12 of the selected 34 articles, it was possible to extract two different experimental groups [27,29,30,31,33,35,41,43,50,54,55,59], giving a final number of 46 experiments. The article information is summarized in Table 1 (general information, materials, and methods) and Table 2 and Table 3 (results on infection prevention and infection treatment, respectively).

#### 3.2.2. Article Quality

**Figure 2:**
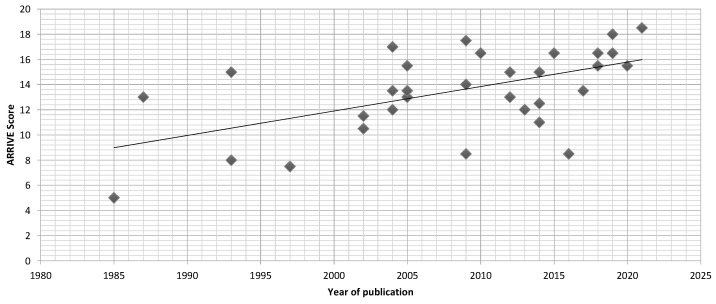
Evolution of ARRIVE scores.

The ARRIVE score varied from 5 to 18.5, and it was possible to identify a trend of higher scores in more recent papers (Figure 2). Five out of thirty-four articles received a negative appreciation [29,35,36,46,59].

#### 3.2.3. Animal Model

**Figure 3:**
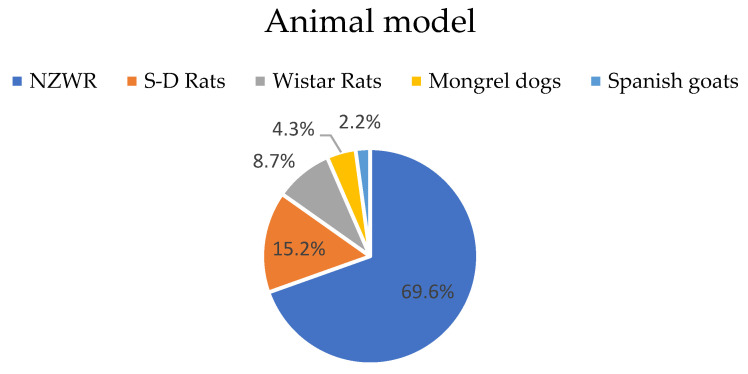
Animal model: percentage of experiments per animal model. NZWR: New Zealand White Rabbit, S–D Rats: Sprague–Dawley Rats.

The New Zealand White rabbit was the most used model in 69.6% of the experiments (32 out of 46) [19,27,29,30,31,32,33,34,35,37,38,39,40,41,42,44,45,47,49,50,52,53,57,58]. Sprague–Dawley rats were used seven times (15.2%) [28,43,46,55,56], Wistar rats were used four times (8.7%) [36,48,54], mongrel dogs were used twice (4.3%) [59], and Spanish goats were used once (2.2%) [51] (Figure 3). The animal populations ranged from 3 to 36 in the experimental groups (mean = 11.04) and ranged from 3 to 25 in the control groups (mean = 9.89).

#### 3.2.4. Type of Bone Defect

All bone defects were created in long bones. The proximal tibia metaphysis was the most used infection site (71.7%), as reported in 33 experiments [29,30,32,33,35,36,37,38,39,40,41,42,43,44,45,46,49,51,52,53,55,56,57,58,59]. The femoral metaphysis was used four times (8.7%): the proximal twice [50] and the distal twice [54]. The diaphysis was used nine times (19,6%): the femur once [48], the tibia four times [28,31,47], and the radius four times [19,27,34]. In this situation, a bone cavity was not produced, rather a contaminated segmental defect was created.

#### 3.2.5. Pathogenic Strain

To induce bone cavity infection or to contaminate the bone defect, *Staphylococcus aureus* was used in all experiments. MRSA [29,30,31,33,37,38,39,41,42,43,44,48,49,52] and MSSA [27,29,30,35,40,45,50,51,53,54,57] were used in 18 (39.1%) and 17 (37.0%) of the experiments, respectively. In 11 series (23.9%), there was no reference to the methicillin resistance of strains [19,28,32,34,36,46,47,55,56,58,59], and this was more frequent in older works. In one paper, the agent was reported to be *S. aureus*, but the ATCC number that was presented does not correspond to any registered strain [34].

#### 3.2.6. Osteomyelitis Induction

To facilitate the development of osteomyelitis, an adjuvant agent was used in 23.9% of the experiments: sodium morrhuate was used in nine [33,37,38,39,40,52,53,57,58] of the series, and arachidonic acid was used in two [35] of the series. In seven experiments (15.2%), the cavities were filled with foreign bodies: metal in five cases [42,43,46,55] (needle in three cases and titanium particles in two cases), PMMA in one case [45], and cotton balls in another case [53]. In 52.2% of the series (24 out of 46), the time to produce osteomyelitis was three weeks [27,31,32,33,37,39,40,41,42,43,44,52,56,59]. In three series (6.5%), it was one week [48,54], while two weeks were needed in five series (10.9%) [35,45,49,58], four weeks were needed in four series (8.7%) [19,36,38,53], and seven weeks were needed in one series (2.2%) [46], which was the only one where the infection was produced in a Sprague–Dawley rat. There was no relation between the time to produce osteomyelitis and the animal model or bacterial agent used. In eight series, the biomaterial was implanted at the same time as the contamination was caused [28,34,47,50,51,55]. In three papers, that information was not available [29,30,57].

#### 3.2.7. Implanted Biomaterial

**Figure 4:**
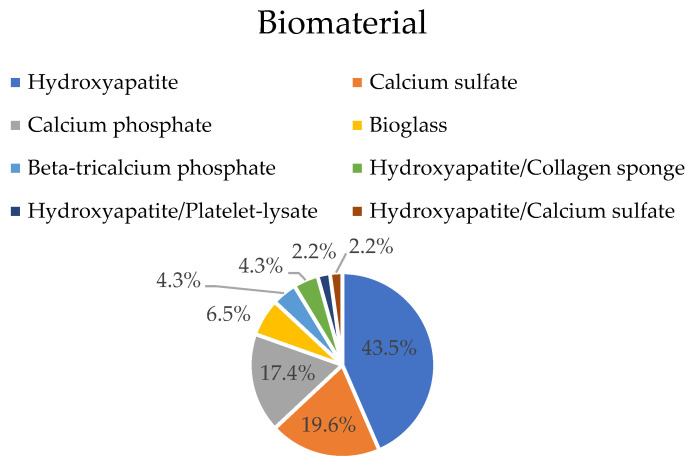
Implanted biomaterial: percentage of experiments per biomaterial.

All the implanted biomaterials were ceramic. Calcium sulfate was present in 19.6% of the experiments (nine groups) [19,27,35,37,42,51,58]. In 33 groups (71.7%), the ceramic was calcium phosphate in different forms: calcium phosphate cement or beads [28,31,33,34,47,48], tricalcium phosphate [43], and hydroxyapatite [29,30,32,36,39,40,41,44,46,49,50,53,54,55,57,59]. In one experiment (2.2%), an association of hydroxyapatite with calcium sulfate was tested [56]. Bioglass was used in three experiments (6.5%) [38,45,52] (Figure 4). The form of the biomaterial is also quite variable: in 22 experiments (47.8%), the biomaterial was used in a pre-formed shape, designed to adapt to the created cavity (cylinders were used in 16 studies [27,29,30,34,37,39,41,42,52,53,55,56,57], pellets were used in 4 studies [19,38,45,51], and blocks were used in 2 studies [36,46]). Other biomaterial forms were used less frequently (34.8%): small pellets or beads were used six times [32,43,45,47,51], powder was used five times [35,44,49,58], granules were used three times [28,59], and sponges were used twice [54], and in eight experiments (17.4%), the material was injected [31,33,35,40,44,48,50].

#### 3.2.8. Antimicrobial Agent

**Figure 5:**
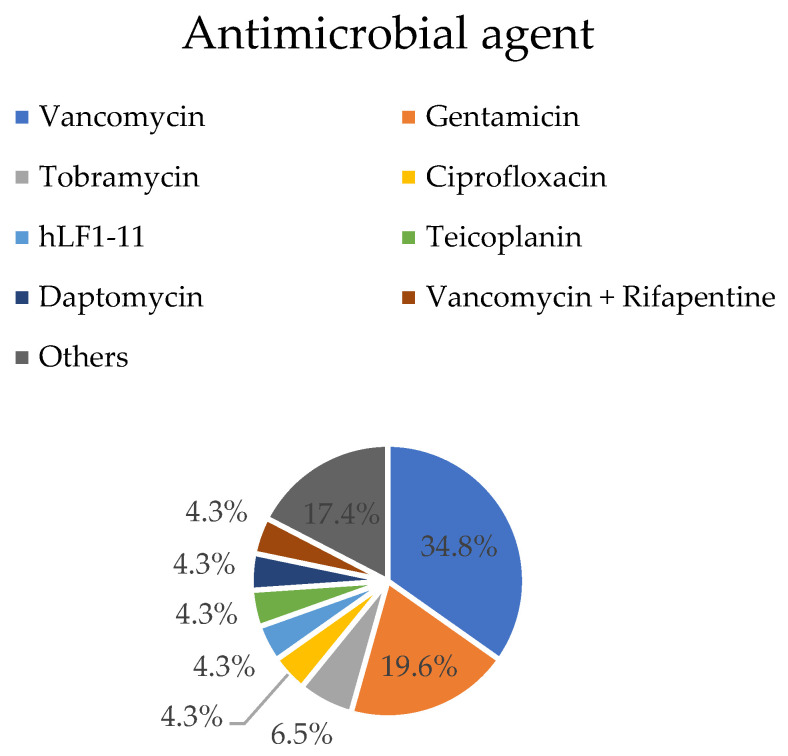
Antimicrobial agent distribution: percentage of studies with each antimicrobial agent. “Others” represent antimicrobial agents used once (2.2%): Povidone-iodine, Flucloxacillin, Arbekacin, Moxifloxacin, Tigecycline, Rifapentine, AMP (antimicrobial peptides), and Cefazolin.

Vancomycin was the antibiotic used in 16 groups (34.8%) [29,30,31,34,39,41,43,49,52,54,57], while gentamicin, the second most frequently applied antimicrobial agent, was used in nine experiments (19.6%) [32,33,35,40,46,50,56,58]. The other tested antibiotics were tobramycin in three experimental groups [19,47,51]; daptomycin [27], teicoplanin [37,38], ciprofloxacin [28,45], and human lactoferrin (hLF1-11) [33,50] in two groups each, and cefazolin [54], rifapentine [53], tigecycline [44], moxifloxacin [42], antimicrobial peptides (AMP) [48], flucloxacillin [59], povidone-iodine [59], and arbekacin [36] in one group each. Rifampicin was used twice in association with vancomycin [55] (Figure 5).

### 3.3. General Results on Infection Evolution and Bone Remodeling

#### 3.3.1. Time to Cure

The time to evaluate the cure of infection and new bone formation ranged from 1 to 26 weeks, with three weeks being the most frequent evaluation time point. Two studies gave no information on the time point to evaluate the infection’s cure or the biomaterial’s osteointegration [30]. Of the 44 studies with this information, 25 had only one time point, which ranged from 1 to 12 weeks [19,27,28,31,33,35,37,38,40,44,45,47,48,49,50,51,52,55,58], with an average of 4.2 weeks. Nineteen studies had more than one time point: seven had two time points [32,41,43,53,56], five had three time points [29,34,54], three had four time points [36,59], three had five time points [39,46,57], and one had six time points [42].

#### 3.3.2. Gross Observation

The general observation of animals was considered in 32 of the 46 experiments (69.6%) [28,29,30,31,32,33,34,35,37,38,39,40,41,44,47,51,52,53,54,55,56,58,59]. Body weight was evaluated in 50.0% of the series (16 out of 32). In addition, the general behavior of the animals, the appearance of sinus tracts and inflammatory signs, co-morbidities, wound healing, and mortality were considered. In 10 out of 16 experimental groups, body weight increased during treatment [30,31,37,38,40,41,52,53,58] while remaining unchanged in 6 experimental groups [31,33,47,56]. Out of 16 control groups, body weight decreased in 8 [30,31,37,38,52,58], remained constant in 7 [33,40,41,47,56], and increased in 1 [53]. Eight studies (17.4%) evaluated the skin characteristics [31,32,34,44,53,55], such as local inflammatory signs, wound dehiscence, or the appearance of sinus tracts. In one study, the healing of wounds was different between groups: in the experimental group, there was complete healing in 10 days, while all animals in the control group showed a failure to heal after five weeks [34]. The appearance of sinus tracts was more frequent in the control group than in the experimental group, although in one series, both groups showed the same complication [31]. The difference in inflammatory signs was reported in one study: they disappeared in the experimental group but remained or increased in the control group [53]. Changes in macroscopic bone morphology were reported in four papers: all animals in the experimental groups evolved to a normal bone shape, in contrast to the controls, where hyperostosis and enlarged bone defects subsided [29,32,54,55]. Two femoral fractures were reported in one experimental group [54], and seven were reported in three control groups [30,54]. Mortality differences were reported in four papers [34,37,38,52]: mortality in the control groups was higher than in the experimental groups.

#### 3.3.3. Blood Tests

Blood tests were used in 12 of the 46 series (26.1%) [29,30,31,32,37,38,40,41,47,53]. In 8 of the 10 experimental groups where the white blood cell (WBC) count was evaluated, there was a decrease after the implantation of the antibiotic-loaded biomaterial, marking a return to normal values [30,31,40,41,47,53]. In two series, the WBC count remained high [31,38]. The variation in WBC count was different in the control groups: one series decreased [47], three series increased [30,37], and the remaining groups remained constant (above normal) [31,40,41,53]. C-reactive protein (CRP) was also studied in five experiments (10.9%) [31,41,53]: it remained above normal levels in all the control groups and decreased to normal levels in two of the experimental groups [31,53]. Other tests were performed in two studies (glutamic-pyruvic transaminase (GPT), glutamic-oxaloacetic transaminase (GOT), urea, and creatinine) without differences between the experimental and control groups [37,38].

#### 3.3.4. Radiological Evaluation

Most selected articles (37 out of 46 studies, representing 80.4%) reported using radiology to evaluate the appearance and evolution of osteomyelitis. The Norden score [60] was used to quantify the status of osteomyelitis in 16 series [29,30,33,35,37,38,39,50,52,53,57], the Aktekin score was used in 2 series [43], and the Beenken score [27], Odekerken score [47], and Dahners score [58] were used in 1 series each; however, in general, there was a subjective appreciation of the radiological bone characteristics. In all but two papers [27,44], there was a radiographic improvement in osteomyelitis in the experimental group. In all the control groups, there was maintenance or increased bone destruction, suggesting osteomyelitis progression. In three series, there was no radiological difference between the experimental and control groups [27,44].

#### 3.3.5. Histological Evaluation

Only seven studies (15.2%) did not undertake a histological evaluation [28,32,35,48,49,51]. The evolution of the histological characteristics of osteomyelitis and the integration of the ceramic material as well as the filling of the bone cavity by new woven bone, increase in collagen, the appearance of new vessels, and signs of inflammation were assessed. The Smeltzer score [61] was used to quantify the histologic pattern and to measure the changes in the osteomyelitis signs in 17 series [29,30,33,37,38,39,40,41,44,50,52,53]. Except for three experiments [27,50], there were differences in the microscopic evaluations between the experimental and control groups, suggesting a favorable evolution after the implantation of the biomaterial with antibiotics.

#### 3.3.6. Microbiological Evaluation

The bacteria present in bone was used to evaluate infection in 33 series (71.7%) [19,27,29,30,32,33,35,37,38,39,40,41,42,43,44,45,46,47,49,51,52,53,54,55,56,58]. The number of positive samples in the experimental groups was lower than that in the control groups in all but one series [27]. A bacteria count was completed in 21 series [27,28,32,33,36,37,42,44,46,47,49,50,51,53,54,55,56,58,62], and although there were differences between the methods used, it was higher in the control groups than in the experimental groups.

## 4. Discussion

This is the first systematic review that summarizes the in vivo effect of adding antibiotics to ceramic bone substitutes to treat experimental osteomyelitis.

### 4.1. Quality of Selected Papers

There was a trend of higher ARRIVE scores in the more recent papers, reflecting the increased care used with animal experiments, namely regarding the number, welfare, and experiment design.

### 4.2. Infection Model

Different animal models were used in the included studies. This may influence the results as, among species, there are differences in bone regeneration and architecture, which poses problems when comparing the results from different studies [63,64]. The New Zealand White rabbit (NZWR) was the most frequently used animal. Rabbits are used in about one-third of all animal musculoskeletal studies [64] due to their relatively low cost, ease of handling, availability, and minimal phylogenetic development [65]. The main drawback is related to size, as rabbits do not allow for large implants [66]. Only two studies used larger animals: mongrel dogs [59] and Spanish goats [51]. Although other animals, such as non-human primates or sheep, should be used because they represent more reliable models, they may pose more ethical issues and limitations in terms of availability, housing, and handling [63,64].

Even for the same species, the location and size of bone defects are variable, leading to different results. The proximal tibia was chosen in most experiments. The resemblance to clinical practice (osteomyelitis occurs mainly around the knee) and easy access justify why it is the first choice. In most series, the infection protocol was based on Norden’s model [67], and the time between inoculation and treatment was three or four weeks. In some series, the antibiotic-loaded biomaterial was implanted simultaneously with bacteria inoculation when the objective was to prevent, rather than cure, bone infection.

There are many infection models, and this may be confusing when comparing different series [68]. Variations between species, the bone defect size and location, the time to introduce the biomaterial, and the existence of a foreign body may explain some of the differences found between studies.

### 4.3. Osteomyelitis Agent

*Staphylococcus aureus* is the most common pathogen isolated in osteomyelitis, which is why it was the chosen agent to develop the experimental infection. Although the incidence of methicillin-resistant *S. aureus* (MRSA) bacteremia has decreased over the past decade [69], MRSA remains associated with poorer clinical outcomes compared with methicillin-sensitive *S. aureus* (MSSA) [70]. Most recent studies tend to use MRSA, reflecting the concern with this agent.

### 4.4. Antibiotics

Gentamicin [32,33,35,40,46,50,56,58] was the antibiotic most often used in earlier studies, but there was a clear shift to vancomycin in most recent research [29,30,31,34,39,41,43,49,52,57]. The use of other antibiotics was scarce, but two papers showed an attempt to develop new substances. In one study, antimicrobial peptides (AMP) were used, originally derived from the venom of the wild bee [48], which seemed capable of avoiding microbial resistance and bypassing the biofilm barrier. In two other studies, human lactoferrin1-11 was used [33,50], a broad-spectrum antibiotic with in vitro activity against both bacteria and fungi that has proven efficacy against MRSA after systemic administration in a mouse model of thigh infection [71]. Although biofilm formation, namely around metallic implants, is responsible for the difficulty in treating implant-related osteomyelitis [72,73], with rifampicin being the antibiotic of choice to overcome its effect [74], only in two series [55] was it used in association with vancomycin. In five series, the antibiotic was encapsulated in poly(lactic-co-glycolic acid) (PLGA) microspheres [29,30,53], which has the advantage of changing the rate and speed of its degradation by adjusting the proportion of microspheres [75]. In one series [31], the antibiotic, together with sodium bicarbonate (NaHCO_3_), was placed in PLGA shells. According to its authors, in the inflammation-induced acidic environment of osteomyelitis, the NaHCO_3_ that was encapsulated in hollow PLGA microspheres rapidly reacted with acid to generate CO_2_ bubbles, disrupting their PLGA shell and thereby promptly releasing the antibiotic. In one series [43], the antibiotic-loaded ceramic material was involved with a poly-l-lactic acid (PLLA) coating, which attempted to slow the antibiotic release [76]. Liposomes were also used as antibiotic carriers in one series [35] based on their beneficial characteristics of being antibiotic and antineoplastic carriers [77]. The dose of the antibiotic added to the biomaterial was very variable between series. In the case of vancomycin, the dose varied between 5% and 20% of the weight. The dose of gentamicin varied between 3.2% and 5% of the weight, but in older series, the value of the antibiotic concentration in the material was not extractable [32,35,46,50,56]. None of the analyzed articles presented results on the effect of different antibiotic concentrations on bone regeneration or considered the eventual toxic effect of antibiotics on osteoblasts under experimental conditions. Many in vitro studies, preliminary to the in vivo studies that were analyzed, presented results on the way the antibiotic was released. Only one article [57] presented the antibiotic release profile in the animal model with infection, an aspect that is crucial not only for the treatment of infection but also, above all, for preventing the emergence of bacterial resistance.

### 4.5. Bioactive Ceramics

Bioactive ceramics are osteoconductive materials (sometimes they may have osteoinductive properties) that are used to fill bone cavities. With time, these ceramics are replaced by new oven bone, behaving as excellent bone substitutes with many advantages over an autologous bone graft [78]. Calcium sulfate and hydroxyapatite were the first ceramics tested. Until 2010, all reviewed papers reported studies on the addition of antibiotics to ceramic material. Most recent papers associate other substances with the ceramic base to promote controlled antibiotic release or enhance new bone formation. Collagen was added in two series [54], benefitting from having a sponge-like elasticity, more easily fitting into bone defects, improving adsorption, and having a long-term release of substances while enhancing bone formation [79].

### 4.6. Evaluation Methods

The general observation of animals showed that the changes in behavior, weight, and fur characteristics, which appeared after osteomyelitis induction, returned to normal in the experimental groups during the first weeks. The number of fractures and deaths in the control groups was superior to that in the experimental groups. Although the evaluated parameters were different between studies, there was an increase in specificity in most recent papers.

The WBC count returned to normal after treatment in all experimental groups and was maintained above normal in the control groups. This was the most reliable laboratory test to reflect the positive evolution after treatment. Other blood tests were not useful due to their low ability to discriminate between the experimental and control groups.

The radiographic evaluation of infection was mainly subjective, qualifying the evolution of the infected cavity, periosteal new bone formation, sequestrum formation, and extent of the disease. In some studies, there was an attempt to quantify and score this evolution. The Norden score was the most-used classification system [60], but other classification systems, as proposed by Beenken [62], Aktekin [80], Odekerken [81], Mader and Wilson [82], and Lane and Sandhu [83], were also used, suggesting that different parameters were being evaluated and scored. These differences in interpretation were a drawback when trying to compare the results between different studies. Other image methodologies were scarcely used (micro-CT in three of the most recent reports [54,55,56] and ^18^F-FDG PET imaging, pQCT imaging, and SEM in one article [45]).

A histological evaluation was used in most studies, using hematoxylin and eosin staining and applying the Smeltzer score [61] based on signs of intraosseous acute inflammation, intraosseous chronic inflammation, periosteal inflammation, and bone necrosis. This score was described to evaluate the development of a murine osteomyelitis model, and, at its origin, there was no introduction of a bone substitute in the infected cavity. The use of this score to evaluate bone behavior in the presence of a bioactive ceramic biomaterial may be inadequate, so new quantification systems are needed.

The microbiological analysis of bone samples was expressed as the presence or absence of bacteria, although some articles gave semi-quantitative results based on the amount of growth in the agar plate. The quantification of CFU on a specific volume of collected bone was made in 20 of the 44 series [27,28,32,33,36,37,42,44,46,47,49,50,51,53,55]. However, it was not possible to conduct a meta-analysis of the quantification of CFU due to the heterogeneity (approximately 80%) in the counting methods and results. Nevertheless, there was an improvement in the experimental groups compared to the control groups.

### 4.7. Infection Treatment and Biomaterial Osteointegration

In all 34 articles, there was some form of an evaluation of the infection evolution. Most of them reported positive results in the experimental group when compared to the control group, suggesting the benefit of adding antibiotics to ceramic materials. The definitions of “good result” and “cure of infection” are variable. Some authors consider the absence of bacterial growth in bone samples [30,32,34,35,37,38,39,40,41,43,45,46,47,51,52,54,55,56], while others value a difference in the CFU count between the experimental and control groups [28,33,36,44,49,50,53]. Of the 46 series, bone swabs or bacteria counts were used in 37 series to evaluate the progression or cure of the infection. Only in 15 experimental groups was there no identification of the bacteria in the bone samples. In the other 22 series, the rate of cure was not total, but it was considered positive if the rate of the cured animals in the experimental group was superior to that of the control group. Only in one article were the results between the experimental and control groups not different [27]. Egawa et al. [54] reported the absence of MSSA in the week 1 bone samples when vancomycin was used and in the week 2 bone samples when cefazolin was added to the bone substitute. In the week 4 bone samples, they reported a decrease in the CFU number in the control group, suggesting that the animal studied (Wistar rat) could cure the infection, even without antibiotics. This report raises the concern that other articles using a similar model may have achieved good results due to the disease’s natural evolution rather than the treatment efficacy. Some authors report good results for the infection evolution, compared to the control groups when the histological or radiographic interpretation reveals a better score. For others, an osteomyelitis cure is considered when no evidence of bacteria is found in the bone samples, and a good result is achieved when the number of animals cured in the experimental group is proportionally higher than that in the control group. Other authors tried to quantify the number of CFUs in a defined bone volume and consider the difference between the groups. The choice of the area of interest is subjective, and the counting methods are different between studies.

Although all papers deal with ceramic materials with the final goal of developing a biomaterial that may be capable of treating infection while promoting bone formation and osteointegration, this was evaluated in only 14 out of 46 series. Cao et al. [29] reported that the scaffold and bone were almost integrated with one another, along with the complete healing of all bone defects in the experimental groups, 12 weeks after implantation. For the same implantation time, Yan et al. [53] reported a large number of type I collagen fibers around the materials, with most of the material degraded and new trabecular bone and cartilage formed. Jia et al. [38], also 12 weeks after implantation, reported that, in the experimental group, newly formed bone was remodeled and restored to its original structural integrity. Xie et al. [52] showed that, 8 weeks after implantation, borate glass was mostly reabsorbed and replaced by new bone. Jiang et al. [39], using hydroxyapatite pellets, suggested that the infected bone became normal bone after 6 weeks, exhibiting reduced periosteal action and a well-shaped trabecular bone structure; however, Koort et al. [45], for the same endpoint, using bioactive glass, could not reach the same conclusion, suggesting the need for longer follow-up time points. Nelson et al. [19] used calcium sulfate pellets and reported that, at four weeks, tobramycin-loaded material showed 96% of the pellets as resorbed and 51% bone formation in the original defect compared to the control group, which showed 71% pellet resorption and 30% bone formation.

Most articles rely on imaging evaluation (histological or radiographic), which is mainly qualitative and observer-dependent, being subjective and introducing bias to the results [84]. Some papers use quantification scores, trying to bring some objectivity to the subjective evaluation, but even those scores, such as the most-used Smeltzer score, were described to evaluate bone infection in the absence of local treatment, which is not the case for the selected articles, and even the selection and definition of the region of interest for analysis are dependent on the observer’s interpretation and judgment [84].

## 5. Conclusions

The addition of an antibiotic to a ceramic biomaterial seemed to be sufficient to make it effective in both the treatment of osteomyelitis and in preventing the evolution of a contaminated bone cavity to osteomyelitis while promoting bone formation and osteointegration for all of the animal models used.

Longer follow-up studies are required to observe the natural evolution of bone infection in animal models: some may have the ability to cure the infection by themselves even in the absence of antibiotics; others, which showed lower bacteria counts for a short follow-up time (but not zero) and were interpreted as a ”good result”, may evolve to chronic infection after the total release of the antibiotic from the biomaterial if the bacteria are still viable.

Appropriate protocols and a standard method of creating bone defects and osteomyelitis are recommended. The New Zealand White rabbit is a very adequate model for this purpose, but the use of a large animal model may be advisable to approximate the results to human pathology. Due to its similarity to human infection, the ideal place to develop a local infection, which was successfully used in most series, is the proximal tibial or distal femoral metaphysis. The use of a local adjuvant agent to create infection is not needed, as the injection of bacterial suspension is enough to develop osteomyelitis. Three weeks is the most adequate time to develop an infection and create a bone cavity without increasing the morbidity and mortality of the animals.

Future studies must evaluate the releasing profile of antibiotics in vivo and under infection. Although the releasing profile of most of the tested biomaterials has been studied in vitro or in healthy animals, there is a lack of knowledge of the drug release profile under conditions closer to reality. This is important data to ensure the absence of low concentrations of antibiotics for long periods in the infected area, which would facilitate the creation of bacterial resistance.

In conclusion, the addition of antibiotics to bioactive ceramic bone substitutes is, apparently, a good solution to treat infected bone cavities while allowing bone regeneration; however, it is not possible to say, as of now, what is the most effective biomaterial for this double purpose. The best way to promote a controlled release, which allows for a concentration above the minimum inhibitory concentration (MIC) for a long enough time to locally eradicate bacteria, is not yet defined. The preferred first-line antibiotic and the ideal ceramic vehicle (pure or in association with other substances) are still under investigation. It is important to follow consistent guidelines and develop appropriate models in order to shorten the amount of time between animal investigation and human application.

## Figures and Tables

**Table 1:** Description of the sample and methods of the selected studies.

Author	ARRIVE	Animal	nE	nC	Agent	Defect Location	T1 (w)	Antibiotic	Material	T2 (w)	Analysis
Eitenmuller [59]	5	Mongrel dogs	6 *	6 *	*S. aureus*	Proximal tibial metaphysis	3	Povidone-iodine	Hydroxyapatite granules	2, 4, 9, and 10	GO; Hist; X-ray
		Mongrel dogs	6 *	6 *	*S. aureus*	Proximal tibial metaphysis	3	Flucloxacillin	Hydroxyapatite granules	2, 4, 9, and 10	GO; Hist; X-ray
Dahners [58]	13	NZWR	10	8	*S. aureus*	Proximal tibial metaphysis	2	Gentamicin	Calcium sulfate	5	GO; Hist; Microb; X-ray
Cornell [32]	15	NZWR	22	9	*S. aureus*	Proximal tibial metaphysis	3	Gentamicin	Hydroxyapatite beads	6 and 17	GO; Microb; X-ray
Korkusuz [46]	8	S-D rats	25	25	*S. aureus*	Proximal tibial metaphysis	7	Gentamicin	Hydroxyapatite	1, 2, 3, 4, and 5	Hist; Microb; X-ray
Itokazu [36]	7.5	Wistar rats	21	21	*S. aureus*	Proximal tibial metaphysis	4	Arbekacin	Hydroxyapatite blocks	1, 3, 5, and 7	Hist; X-ray
Nelson [19]	11.5	NZWR	13	13	*S. aureus*	Radial diaphysis	4	Tobramycin	Calcium sulfate pellets	4	Hist; Lab; Microb; X-ray
Shirtliff [49]	10.5	NZWR	12	10	MRSA	Proximal tibial metaphysis	2	Vancomycin	Hydroxyapatite cement	4	Microb; X-ray
Buxton [28]	12	S-D rats	6	6	*S. aureus*	Tibia diaphysis	0	Ciprofloxacin (1)	Calcium phosphate cement	2	GO; Lab
Joosten [40]	13.5	NZWR	11	6	MSSA	Proximal tibial metaphysis	3	Gentamicin	Hydroxyapatite cement	3	GO; Hist; Lab; Microb; X-ray
Stallmann [50]	17	NZWR	7	8	MSSA	Proximal femur	0	hLF1-11	Hydroxyapatite cement	3	Hist; Lab; X-ray
		NZWR	6	8	MSSA	Proximal femur	0	Gentamicin	Hydroxyapatite cement	3	Hist; Lab; X-ray
Faber [33]	15.5	NZWR	8	6	MRSA	Proximal tibial metaphysis	3	Gentamicin	Calcium phosphate cement	3	GO; Hist; Lab; Microb; X-ray
		NZWR	8	6	MRSA	Proximal tibial metaphysis	3	hLF1-11	Calcium phosphate cement	3	GO; Hist; Lab; Microb; X-ray
Joosten [41]	13.5	NZWR	6	5	*S. aureus* SCV	Proximal tibial metaphysis	3	Vancomycin	Hydroxyapatite cement	3 and 6	GO; Hist; Lab; Microb
		NZWR	7	6	MRSA	Proximal tibial metaphysis	3	Vancomycin	Hydroxyapatite cement	3 and 6	GO; Hist; Lab; Microb
Koort [45]	13.5	NZWR	9	5	MSSA	Proximal tibial metaphysis	2	Ciprofloxacin	Microspheres of bioactive glass (2)	6	Hist; Microb; PET; pQCT; SEM; X-ray
Thomas [51]	13	Spanish goats	12	12	MSSA (3)	Proximal tibial metaphysis	0	Tobramycin	Calcium sulfate pellets	3	GO; Microb; X-ray
Hui [35]	8.5	NZWR	6	6	MSSA	Proximal tibial metaphysis	2	Gentamicin (4)	Calcium sulfate	2	GO; Microb; X-ray
		NZWR	6	6	MSSA	Proximal tibial metaphysis	2	Gentamicin	Calcium sulfate	2	GO; Microb; X-ray
Kanellakopoulou [42]	14	NZWR	36	18	MRSA	Proximal tibial metaphysis	3	Moxifloxacin	Calcium sulfate (5)	1, 2, 3, 4, 5, and 6	Hist; Microb
Xie [52]	17.5	NZWR	16	11	MRSA	Proximal tibial metaphysis	3	Vancomycin	Borate glass pellets	8	GO; Hist; Lab; Microb; X-ray
Jia [37]	16.5	NZWR	12	12	MRSA	Proximal tibial metaphysis	3	Teicoplanin	Calcium sulfate paste	6	GO; Hist; Lab; Microb; X-ray
Jia [38]	16.5	NZWR	14	14	MRSA	Proximal tibial metaphysis	4	Teicoplanin	Borate glass pellets	12	GO; Hist; Lab; Microb; X-ray
Jiang [39]	15	NZWR	20	20	MRSA	Proximal tibial metaphysis	3	Vancomycin	Nanohydroxyapatite pellets	1, 2, 3, 6, and 12	GO; Hist; Microb; X-ray
Kaya [44]	13	NZWR	7	7	MRSA	Proximal tibial metaphysis	3	Tigecycline	Calcium hydroxyapatite cement	3	GO; Hist; Microb; X-ray
Huang [34]	12	NZWR	12	12	*S. aureus* (6)	Radial diaphysis	0	Vancomycin	Calcium phosphate cement (7)	4, 8, and 12	GO; Hist; X-ray
Beenken [27]	11	NZWR	6	6	MSSA	Radial diaphysis	3	Daptomycin	Calcium sulfate hemihydrate	3	Hist; Microb; X-ray
		NZWR	6	6	MSSA	Radial diaphysis	3	Daptomycin	Calcium sulfate (8)	3	Hist; Microb; X-ray
Chung [31]	12.5	NZWR	6	6	MRSA	Tibia	3	Vancomycin (9)	Calcium phosphate cement	3	GO; Hist; Lab
		NZWR	6	6	MRSA	Tibia	3	Vancomycin	Calcium phosphate cement	3	GO; Hist; Lab
Kankilic [43]	15	S-D rats	10	12	MRSA	Proximal tibial metaphysis	3	Vancomycin	β-tricalcium phosphate (10)	1 and 6	Hist; Microb; X-ray
		S-D rats	10	12	MRSA	Proximal tibial metaphysis	3	Vancomycin (coated)	β-tricalcium phosphate (10)	1 and 6	Hist; Microb; X-ray
Yan [53]	16.5	NZWR	8	8	MSSA	Proximal tibial metaphysis	4	Rifapentine (11)	Bone-like hydroxyapatite scaffold (12)	4 and 12	GO; Hist; Lab; Microb; X-ray
Cao [29]	8.5	NZWR	5	5	MSSA	Proximal tibial metaphysis	(13)	Vancomycin (11)	Hydroxyapatite scaffold (12)	4, 8, and 12	GO; Hist; Lab; Microb; X-ray
		NZWR	5	5	MRSA	Proximal tibial metaphysis	(13)	Vancomycin (11)	Bone-like hydroxyapatite (12)(14)	4, 8, and 12	GO; Hist; Lab; Microb; X-ray
Cao [30]	13.5	NZWR	12	12	MSSA	Proximal tibial metaphysis	(13)	Vancomycin	Bone-like hydroxyapatite (12)	NA	GO; Hist; Lab; Microb; X-ray
		NZWR	12	12	MRSA	Proximal tibial metaphysis	(13)	Vancomycin	Bone-like hydroxyapatite (12)	NA	GO; Hist; Lab; Microb; X-ray
Lulu [47]	15.5	NZWR	4	4	*S. aureus*	Tibia midshaft	0	Tobramycin	Calcium phosphate beads	4	GO; Hist; Lab; Microb; X-ray
Melicherčík [48]	16.5	Wistar rats	8	8	MRSA	Femoral cavities	1	AMP	Calcium phosphate	1	X-ray
Egawa [54]	18.0	Wistar rats	18	18	MSSA	Distal femur	1	Cefazolin	Hydroxyapatite/collagen sponge	1, 2, and 4	GO; Hist; Micro-CT, Microb
		Wistar rats	18	18	MSSA	Distal femur	1	Vancomycin	Hydroxyapatite/collagen sponge	1, 2, and 4	GO; Hist; Micro-CT, Microb
Hasan [55]	16.5	S-D rats	5	12	*S. aureus*	Proximal tibial metaphysis	0	Vancomycin and rifampicin	Hydroxyapatite (15)	10	GO; Hist; Microb; X-ray
		S-D rats	3	3	*S. aureus*	Proximal tibial metaphysis	0	Vancomycin and rifampicin	Hydroxyapatite (15)	6	GO; Hist; Microb; X-ray
Liu [57]	15.5	NZWR	10	10	MSSA	Proximal tibial metaphysis	(13)	Vancomycin	Platelet-lysate/nano-hydroxiapatite	1, 2, 3, 6, and 12	Hist; X-ray
Dvorzhinskiy [56]	18.5	S-D Rats	32	20	*S. aureus*	Proximal tibial metaphysis	3	Gentamicin	Hydroxyapatite/CaS	6 and 26	GO; Hist; Micro-CT; Microb

nE: number of animals in experimental group; nC: number of animals in control group; T1 (w): time to induce osteomyelitis (weeks); T2 (w): time between bioceramic implantation and endpoint (weeks); NZWR: New Zealand White rabbit; S–D rats: Sprague–Dawley rats; GO: gross observation; Hist: histhology; Lab: laboratory blood tests; Microb: microbiological tests; PET: positron emission tomography; pQCT: peripheral quantitative computed tomography; Micro-CT: micro-computed tomography; MRSA: methicillin-resistant *Staphylococcus aureus*; MSSA: methicillin-sensitive *Staphylococcus aureus*; SCV: small colony variant; PLGA: poly(D,L-lactic-co-glycolic acid); NA: data not available; AMP: antimicrobial peptides (AMP) consisting of 12 amino acid residues (H-Gly-Lys-Trp-Met-Lys-Leu-Leu-Lys-Lys-Ile-Leu-Lys-NH2). (*) Each animal was subjected to the preparation of three infected cavities. One was treated with the biomaterial with antibiotic while in the other two, the same material without antibiotics was used. The same animal thus belonged to both the experimental and control groups. (1) linked to methyldiphosphonate; (2) linked to Racemic poly(DL)-lactide (PDLLA); (3) resistant to streptomycin-modified MSSA; (4) lipossomal; (5) synthetic crystallic semihydrate form; (6) ATCC28923 subspecies reported in this paper does not correspond to *S. aureus spp*; (7) containing 2 mg of icariin; (8) coated with chitosan; (9) pH-responsive hollow PLGA microspheres; (10) with poly-L-lactic acid; (11) loaded PLGA microspheres; (12) with poly(amino acid); (13) information not available (original paper refers to another paper published in Chinese); (14) with incorporated Poly(lactic-co-glycolic) acid (PLGA) microspheres; (15) over a calcium carbonate core.

**Table 2:** Summary of general results on infection prevention and bone remodeling for experimental groups and for antibiotic and biomaterial used.

Author	Antibiotic Characteristics			Ceramic Used			Discussion	
	Antibiotic Used	Formulation	Amount	Material	Form	Amount	Infection	Bone Formation
Buxton [28]	Ciprofloxacin	E41 (1)	0.35% wt	Calcium phosphate	Granules	0.1 mL (2)	Fewer bacteria; no gross signs of osteomyelitis	Histological evidence of bone healing
Stallmann [50]	hLF1-11 (3)	NI	5% wt	Calcium phosphate	Injectable cement	NI	Significant decrease in viable bacteria	Not different from the control group
	Gentamicin	NI	5% wt	Calcium phosphate	Injectable cement	NI	Significant decrease in viable bacteria	Better remodeling by ingrowing bone
Thomas [51]	Tobramycin	Powder	10% wt	Calcium sulfate	Pellets	Fifteen pellets/animal	Prevented infection in 10/12 animals	NI
Huang [34]	Vancomycin	Solution (4)	2 mg IC and 20 mg vancomycin per cylinder	Calcium phosphate	Cylinders (4 mm × 15 mm)	One cylinder/animal	No bacteria were detected; all controls showed signs of infection	Defects were completely repaired by the 12th week; all controls showed progression of bone destruction
Lulu [47]	Tobramycin	Solution	NI (5)	Calcium phosphate	0.2 g beads	One bead/animal	Inhibition of the *S. aureus* growth	Repaired bone defect and recanalization of the medullary cavity
Hasan [55]	Vancomycin and rifampicin	Solution	NI (6)	Hydroxyapatite over a calcium carbonate core	Cylinders (4 mm diameter × 3.5 mm height)	One cylinder/animal	Disappearance of all clinical, imagological, and microbiological signs of infection	Healed bone with cortical bridging, new bone growth, and osseointegration
	Vancomycin and rifampicin	Solution	NI (6)	Hydroxyapatite over a calcium carbonate core	Cylinders (4 mm diameter × 3.5 mm height)	One cylinder/animal	Healing without any signs of infection	New bone formation, ongoing bridging of newly formed bone, and limited mature collagen structure

NI: No information. (1) Dry powder of methyl bisphosphonate covalently linked to ciprofloxacin; (2) estimated; (3) human lactoferrin 1–11; (4) IC: 8 mg/mL ethanol and vancomycin: 80 mg/mL PBS; (5) beads were dipped in 30 mg/mL tobramycin solution for 24 h; (6) 350 mg of ceramic particles were soaked with agitation (100 rpm) in a 1 mL solution of 80 mg/mL of vancomycin and 20 mg/mL rifampicin, in 50:50 water:DMSO solvent.

**Table 3:** Summary of general results on infection evolution and bone remodeling for experimental groups and for antibiotic and biomaterial used.

Author	Antibiotic Characteristics			Ceramic Used			Discussion	
	Antibiotic Used	Formulation	Amount	Material	Form	Amount/Animal	Infection	Bone Formation
Dahners [58]	Gentamicin	Powder	50 mg per cm^3^ of calcium sulfate	Calcium sulfate	Powder	1 cm^3^	Cure rate of 2/10; clinical and radiographic improvement in all other animals	NI
Eitenmuller [59]	Povidone-iodine	NI	10% wt	Hydroxyapatite	Granules	NI	Resolution of clinical and radiological signs of infection	Good osteointegration of material at 10 weeks
	Flucloxacillin	NI	10% wt	Hydroxyapatite	Granules	NI	Resolution of clinical and radiological signs of infection	Only peripheral osteointegration of material at 10 weeks
Cornell [32]	Gentamicin	Gentamicin sulfate and gentamicin crobefat	NI	Hydroxyapatite	Beads	40 mg	Infection eradication in 16/22 animals	NI
Korkusuz [46]	Gentamicin	Powder	5 mg/block	Hydroxyapatite	Blocks (4 × 3 × 3 mm)	One block	Eradication of infection without removal of the metal implants in all animals	NI
Itokazu [36]	Arbekacin	Powder	0.84 mg/block	Hydroxyapatite	Blocks (2 × 2 × 3 mm)	One block	Cure in 5/7 rats	New bone formation was visible at the surface of the block and complete contact without fibrous tissue was evident at the interface between the bone and implant at 7 weeks
Nelson [19]	Tobramycin	Powder	10% wt	Calcium sulfate	Pellets (3.4 mm diameter × 4.7 mm length, average weight of 100 mg)	Six pellets (average)	Infection cure in 11/13 animals	Rabbits showed 96% of the pellets resorbed and 51% bone formation in the original defect
Shirtliff [49]	Vancomycin	Powder	10% wt	Hydroxyapatite	Powder	2–7 g	Infection cure rate of 81.8%	NI
Joosten [40]	Gentamicin	Powder	3.2% wt	Hydroxyapatite (1)	Paste	1.4–2.5 g (average 2.0 g)	No evidence of infection in all animals	Little evidence of resorption
Faber [33]	Gentamicin	Powder	5% wt	Calcium phosphate (2)	Paste	2.4 ± 0.3 g	Absence of bacteria in 6/8 animals; imagiological signs of infection present in 5/8	NI
	hLF1-11	NI	5% wt	Calcium phosphate (2)	Paste	2.2 ± 0.2 g	Infection cure in 5/8 animals; significantly reduced bacterial load in 2/8	NI
Joosten [41]	Vancomycin	Powder	16% wt	Hydroxyapatite (1)	Cylinders (6 mm diameter × 12 mm length)	NI	No evidence of infection in all animals	Little evidence of resorption
	Vancomycin	Powder	16% wt	Hydroxyapatite (1)	Cylinders (6 mm diameter × 12 mm length)	NI	No evidence of infection in all animals	Little evidence of resorption
Koort [45]	Ciprofloxacin	Powder	7.6% wt	Bioactive glass (3)	Pellets (1 mm diameter × 0.9 mm length)	NI	Successful for eradication of the bone pathogen; soft tissue infections need systemic antimicrobial treatment	Need to perform a long-term follow-up of the osteoconductive response
Hui [35]	Gentamicin	Loaded liposomes	NI	Calcium sulfate	Powder	NI	Complete sterilization of bone (100% cure)	NI
	Gentamicin	Powder	NI	Calcium sulfate	Powder	NI	More effective than controls, but did not sterilize all bone tissues	NI
Kanellakopoulou [42]	Moxifloxacin	Powder	10% wt	Calcium sulfate	Cylinder (50 mg)	One cylinder	Complete eradication of infection	NI
Xie [52]	Vancomycin	Powder	8% wt	Borate glass	Pellets (6 mm × 6 mm)	NI	Treatment rate of 73.3%	Borate glass mostly reabsorbed and replaced by new bone
Jia [37]	Teicoplanin	Powder	10% wt	Calcium sulfate	Pellets (4.7 mm diameter × 3.5 mm length)	NI	Lower radiological and histological scores and lower rate of MRSA culture, but did not resolve bone infection in all animals	Newly formed bone remodeled and restored to its original structural integrity
Jia [38]	Teicoplanin	Powder	8% wt	Borate glass (4)	Pellets (4.7 mm diameter × 3.5 mm length)	NI	Lower rate of MRSA culture	Degradation of pellets and new bone formation
Jiang [39]	Vancomycin	Powder	16% wt	Nanohydroxyapatite	Cylinders (3.2 mm diameter × 10 mm length)	NI	Bacteria count decreased significantly	Normal bone after 12 weeks
Kaya [44]	Tigecycline	Powder	5% wt	Hydroxyapatite	Powder	0.5–2 g	Decline in all clinical and imagological signs of infection	NI
Beenken [27]	Daptomycin	Powder	15% wt	Calcium sulfate	Cylinders (4 mm diameter × 10 mm length)	One cylinder	Reduction in bacteria count was not different from controls	NI
	Daptomycin	Powder	15% wt	Calcium sulfate (5)	Cylinders (4 mm diameter × 10 mm length)	One cylinder	Significant reduction in bacteria count	NI
Chung [31]	Vancomycin	Shells (6)	20% wt	Calcium phosphate	Paste	NI	Highly effective local antibacterial activity	NI
	Vancomycin	Powder	5% wt	Calcium phosphate	Paste	NI	Reduction in inflammation signs	NI
Kankilic [43]	Vancomycin	Beads	10% wt	Calcium phosphate (7)	1.5 mm diameter beads	NI	Cure of infection in all animals	Biocompatibility and osteointegration
	Vancomycin	Coated beads	10% wt	Calcium phosphate (7)	PLLA-coated 1.5 mm diameter beads	NI	Cure of infection in all animals	Biocompatibility and osteointegration
Yan [53]	Rifapentine	Microspheres (8)	4% wt	Hydroxyapatite (9)	Cylinders (5 mm diameter × 15 mm length)	One cylinder	Bacterial colony counts were extremely low, suggesting eradication of infection	Most of the material was degraded and new trabecular bone formed; bone shape gradually improved and returned to normal
Cao [29]	Vancomycin	Microspheres (10)	8% wt	Hydroxyapatite (9)	Cylinders (5 mm diameter × 15 mm length)	3 g	Progressive disappearance of imagological signs of infection	Scaffold almost integrated with complete healing of all bone defects
	Vancomycin	Microspheres (10)	8% wt	Hydroxyapatite (9)	Cylinders (5 mm diameter × 15 mm length)	3 g	Progressive disappearance of imagological signs of infection	Scaffold almost integrated with complete healing of all bone defects
Cao [30]	Vancomycin	Microspheres (10)	8% wt	Hydroxyapatite (9)	Cylinders (5 mm diameter × 15 mm length)	3 g	Curative ratio reached 75%	NI
	Vancomycin	Microspheres (10)	8% wt	Hydroxyapatite (9)	Cylinders (5 mm diameter × 15 mm length)	3 g	Curative ratio reached 66.7%	NI
Melicherčík [48]	Antimicrobial peptides (AMP)	NI	5% wt	Calcium phosphate	Paste	NI	Reduced infection	Minimal signs of the presence of the carrier, probably as a result of its resorption
Egawa [54]	Cefazolin	Powder	2% wt	Hydroxyapatite (11)	Sponges (3 × 3 × 4 mm)	One sponge	MSSA proliferation was prevented at week 2	Some degradation of ceramic, without complete osteointegration
	Vancomycin	Powder	2% wt	Hydroxyapatite (11)	Sponges (3 × 3 × 4 mm)	One sponge	MSSA proliferation was prevented at week 1	Implanted material was maintained and replaced with new bone at week 4
Liu [57]	Vancomycin	Powder	16% wt	Hydroxyapatite	Cylinders (6 mm diameter × 20 mm length)	One cylinder	Progressive disappearance of radiographic and histological signs of infection	Lamellar bone was formed
Dvorzhinskiy [56]	Gentamicin	Powder	0.29 mg (12)	Hydroxyapatite/calcium sulfate	Cylinders (3 mm diameter × 3 mm length)	One cylinder	No infection was detectable at both 6 weeks and 6 months	New bone growth was detected

NI: No information. (1) Equimolar mixture of amorphous calcium phosphate and dicalcium phosphate; (2) composition of 62.5% α-TCP, 26.8% dicalcium phoshate dihydrate, 8.9% calcium carbonate, and 1.8% precipitated hydroxyapatite; (3) racemic poly(DL)-lactide (PDLLA) and microspheres of bioactive glass; (4) pellets of borate glass/chitosan composite; (5) with chitosan coating; (6) shells of poly(D,L-lactic-co-glycolic acid) (PLGA) and aqueous cores of vancomycin and sodium bicarbonate (NaHCO_3_); (7) BetaTCP mixed with PLLA; (8) rifapentine-encapsulated poly(lactic-co-glycolic acid) microspheres; (9) hydroxyapatite/poly amino acid (BHA/PAA) scaffold; (10) vancomycin-encapsulated poly(lactic-co-glycolic acid) microspheres; (11) with collagen; (12) 0.29 mg per cylinder, though cylinder weight not available.

## Data Availability

Data are available upon request.
